# Three *Pseudomonas putida* FNR Family Proteins with Different Sensitivities to O_2_*

**DOI:** 10.1074/jbc.M115.654079

**Published:** 2015-05-13

**Authors:** Susan A. Ibrahim, Jason C. Crack, Matthew D. Rolfe, José Manuel Borrero-de Acuña, Andrew J. Thomson, Nick E. Le Brun, Max Schobert, Melanie R. Stapleton, Jeffrey Green

**Affiliations:** From the ‡ Krebs Institute, Molecular Biology and Biotechnology, University of Sheffield, Sheffield, S10 2TN, United Kingdom,; the § Centre for Molecular and Structural Biochemistry, School of Chemistry, University of East Anglia, Norwich, NR4 7TJ, United Kingdom, and; ¶ Institut für Mikrobiologie, Technische Universität, D-38106 Braunschweig, Germany

**Keywords:** bacterial signal transduction, bacterial transcription, iron-sulfur protein, microbiology, transcription factor, transcription regulation

## Abstract

The *Escherichia coli* fumarate-nitrate reduction regulator (FNR) protein is the paradigm for bacterial O_2_-sensing transcription factors. However, unlike *E. coli*, some bacterial species possess multiple FNR proteins that presumably have evolved to fulfill distinct roles. Here, three FNR proteins (ANR, PP_3233, and PP_3287) from a single bacterial species, *Pseudomonas putida* KT2440, have been analyzed. Under anaerobic conditions, all three proteins had spectral properties resembling those of [4Fe-4S] proteins. The reactivity of the ANR [4Fe-4S] cluster with O_2_ was similar to that of *E. coli* FNR, and during conversion to the apo-protein, via a [2Fe-2S] intermediate, cluster sulfur was retained. Like ANR, reconstituted PP_3233 and PP_3287 were converted to [2Fe-2S] forms when exposed to O_2_, but their [4Fe-4S] clusters reacted more slowly. Transcription from an FNR-dependent promoter with a consensus FNR-binding site in *P. putida* and *E. coli* strains expressing only one FNR protein was consistent with the *in vitro* responses to O_2_. Taken together, the experimental results suggest that the local environments of the iron-sulfur clusters in the different *P. putida* FNR proteins influence their reactivity with O_2_, such that ANR resembles *E. coli* FNR and is highly responsive to low concentrations of O_2_, whereas PP_3233 and PP_3287 have evolved to be less sensitive to O_2_.

## Teaser

**Background:** FNR proteins are O_2_-responsive bacterial transcription factors.

**Results:** *Pseudomonas putida* possesses three FNR proteins with iron-sulfur clusters that have different sensitivities to O_2_.

**Conclusion:** The mechanism of the iron-sulfur cluster reaction with O_2_ is conserved among FNR proteins.

**Significance:** Differential sensitivity of multiple FNR proteins extends the range of O_2_-responsive gene expression within a single bacterium.

## Introduction

Fumarate-nitrate reduction regulator (FNR)^2^ proteins are a major subgroup of the cyclic-AMP receptor protein family of bacterial transcription regulators (1). The major function of FNR proteins is the reprogramming of gene expression to coordinate the switch from aerobic to anaerobic metabolism when facultative anaerobes like *Escherichia coli* are starved of O_2_ (2–7). The paradigm for O_2_-sensing transcription factors is the *E. coli* FNR protein. The N-terminal region of FNR contains four essential cysteine residues that coordinate an O_2_-sensitive [4Fe-4S] cluster (8, 9). In the absence of O_2_, the [4Fe-4S] cluster is stable, and FNR exists as a homodimer that is capable of high affinity, site-specific DNA binding to an FNR box (TTGATNNNNATCAA) (9, 10). When bound to target DNA, FNR activates the expression of genes encoding proteins required for anaerobic metabolism and represses those utilized under aerobic conditions (2, 4), such that when O_2_ is available, anaerobic metabolism is shutdown in favor of the more energetically efficient aerobic respiratory metabolism. Molecular oxygen reacts with the FNR [4Fe-4S] cluster in a series of steps that ultimately yields the apo form of the protein (Equations 1–3) (11–13).

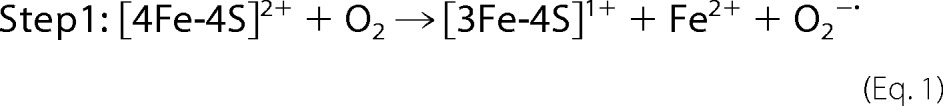


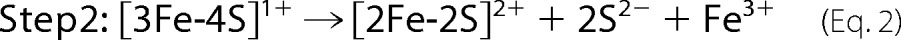


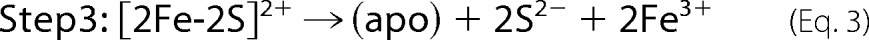
 Recent work has shown that step 2 (Equation 2) is more complex than previously envisaged because it involves the conversion of the [3Fe-4S]^1+^ cluster to a persulfide-coordinated [2Fe-2S]^2+^ form. The [4Fe-4S] to [2Fe-2S] conversion can therefore be written as in Equation 4 (14).

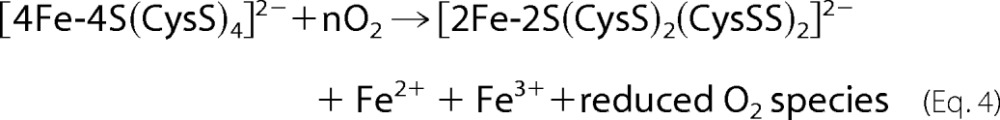
 The retention of cluster sulfide (as CysSS) permits facile repair of the FNR [4Fe-4S] cluster in the presence of Fe^2+^ and a reducing agent (14). Molecular oxygen-dependent conversion of the [4Fe-4S] cluster to the persulfide-ligated [2Fe-2S] causes conformational rearrangements at the dimer interface that result in a charge-clash in the dimerization helices (15). Consequently, FNR monomerizes and is no longer able to bind DNA or to regulate transcription (10). Upon prolonged exposure to O_2_ *in vitro* and *in vivo*, the dominant FNR species is the monomeric apo form, which is capable of acquiring a [4Fe-4S] cluster via the action of the Isc (iron-sulfur cluster) biosynthetic machinery, such that FNR continually monitors the cytoplasm for the availability of O_2_ (16–18).

Unlike many bacteria, *Pseudomonas putida* and *Burkholderia* spp. possess multiple FNR family proteins that retain the characteristic cluster of cysteine residues in the N-terminal sensory domain. Transcriptomic analysis of the opportunistic pathogen *Burkholderia cenocepacia* revealed the presence of a 50-gene low oxygen-activated (*lxa*) locus that was associated with persistence of this obligate aerobe under anaerobic conditions (19). The *lxa* locus includes the FNR protein BCAM0287, which was induced 17-fold under low O_2_ (microaerobic) conditions. In addition, two other FNR protein encoding genes were induced during growth under a 6% O_2_ atmosphere, BCAM0049 (induced 77-fold, compared with aerobic conditions) and BCAM1483 (induced 3.5-fold) (19). Although an FNR box-like motif was associated with many genes induced under microaerobic conditions, the functions of the multiple FNR regulators in *B. cenocepacia* are poorly defined. Similarly, the properties and functions of the three FNR proteins of *P. putida* KT2440 are poorly understood. Here for the first time, three FNR proteins (PP_3233, PP_3287, and PP_4265, the last of which is also known as ANR) from a single bacterial species, *P. putida* KT2440, have been isolated, and their responses to O_2_ *in vivo* and *in vitro* have been assessed.

## Experimental Procedures

### 

#### 

##### Overproduction and Purification of Proteins

The ANR open reading frame was amplified from *P. putida* KT2440 genomic DNA using the primers MS87 (5′-TTTTTCTAGACATGTCCGAGCCAGTCAAACT-3′) and MS88 (5′-TTTTCTCGAGTCAGGCCTCGATTGCACCACA-3′) containing engineered XbaI and XhoI sites, respectively, and ligated into pGEX-KG to give construct pGS2268 (see Table 1). The resulting GST-ANR fusion protein was overproduced following IPTG induction in aerobic cultures of *E. coli* BL21 harboring pGS2268 for 2 h at 37 °C. The fusion protein was purified from cell-free extracts using glutathione-Sepharose 4B (GE Healthcare) equilibrated with 25 mm HEPES, 100 mm NaCl, 100 mm NaNO_3_, 2.5 mm CaCl_2_ at pH 7.5. The ANR protein was released from the fusion protein by on-column thrombin cleavage. The ANR, PP_3233 and PP_3287 open reading frames were amplified from *P. putida* KT2440 genomic DNA and ligated into the pCOLD-TF vector (Takara Clontech) (to give pGS2414, pGS2403, and pGS2413, respectively) using the following primers: ANR, MS140 (5′-TTTTCATATGTCCGAGCCAGTCAAACTGCG-3′) and MS150 (5′-TTTTTCTAGATCAGGCCTCGATTGCACCAC-3′) containing engineered NdeI and XbaI sites, respectively; PP_3233, MS141 (5′-TTTTCATATGTCAGGCTCTGCAGAAATGGG-3′) and MS92 (5′-TTTTCTCGAGTCAAGTGGGCTCCTCCAGGC-3′) containing engineered NdeI and XhoI sites, respectively; PP_3287, MS142 (5′-TTTTCATATGCCTGGCCAGCTGAAGGTCAC-3′) and MS151 (5′-TTTTTCTAGATCAGGGGCCCTTGGCCTCAC-3′) containing engineered NdeI and XbaI sites, respectively. The resulting His_6_-Tig-ANR, -PP_3233, and -PP_3287 fusion proteins were overproduced in aerobic cultures of *E. coli* BL21 harboring pGS2414, pGS2403, or pGS2413, respectively, for 24 h at 15 °C. The fusion proteins were purified from cell-free extracts on nickel-charged Hi-Trap chelating columns (GE Healthcare) equilibrated with 20 mm sodium phosphate, 0.5 m NaCl, pH 7.4, and were eluted by application of a linear imidazole gradient (0–1 m) followed by desalting into 25 mm HEPES, 100 mm NaCl, 100 mm NaNO_3_, pH 7.5. Protein concentration was determined by the Bio-Rad protein reagent (20).

##### Gel Filtration, Protein Reconstitution, and Total Amino Acid Analysis

The oligomeric state of ANR was estimated from the elution volume of a sample (50 μl of 250 μm protein) applied to a calibrated Superdex 200 column. The column was equilibrated with 25 mm Tris-HCl, pH 7.5, containing 500 mm NaCl and 2 mm EDTA, and the standards used to calibrate the column were blue dextran, hemoglobin, ovalbumin, cytochrome *c*, and aprotinin.

The ANR, PP_3233, and PP_3287 proteins were reconstituted overnight under anaerobic conditions at 25 °C in 25 mm HEPES, 100 mm NaCl, 100 mm NaNO_3_, pH 7.5, to which 0.5 mm l-cysteine, 12.5 mm DTT, an 8-fold molar excess of (NH_4_)_2_Fe(SO_4_)_2_ and 0.2 μm NifS cysteine desulfurase were added. Reconstituted proteins were purified on a heparin column (GE Healthcare) and eluted in 25 mm Tris-HCl containing 500 mm NaCl, pH 7.5 (21). Iron content was determined as previously described (21). Total amino acid analysis was carried out by Alta Bioscience (University of Birmingham, Birmingham, UK) following complete acid hydrolysis of ANR protein samples that had previously had the protein content estimated by the Bio-Rad protein assay (20).

##### UV-visible and CD Spectroscopy

Sealed anaerobic cuvettes containing reconstituted FNR proteins were injected with increasing amounts of air-saturated buffer as indicated and were incubated at 25 °C for 10 min followed by spectroscopic analysis. Absorbance measurements were made with a Cary UV-visible spectrophotometer. Changes in absorbance at 405 nm were used to monitor the conversion of the clusters. The extinction coefficient for the *E. coli* FNR iron-sulfur cluster (ϵ_406 nm_ = 16,200 m^−1^ cm^−1^) was used to calculate the amount of [4Fe-4S]^2+^ cluster in the reconstituted proteins. The spectra shown are typical of at least three experiments. CD measurements were made with a Jasco J-810 spectropolarimeter. Aliquots of ANR (680 μl) were diluted to 29.8 μm iron-sulfur cluster under anaerobic conditions for initial measurements before treating with oxygenated buffer (∼220 μm O_2_) to give ∼2-fold molar excess O_2_ and then incubated for 15 min at room temperature prior to further measurements.

##### Kinetic Measurements

Reactions were initiated by the injection of air-saturated buffer (final concentration, ∼100 μm O_2_) into sealed anaerobic cuvettes containing reconstituted ANR, PP_3233, or PP_3287 proteins (final concentration, ∼6–9 μm [4Fe-4S]) at 25 °C. The dead time of mixing was ∼5 s. Changes in absorbance at 420 nm were used to monitor the conversion of the clusters. The A_420 nm_ decay data were fitted to a single or double exponential function (as appropriate) using the program Origin (version 8; OriginLab). Where a double exponential function was fitted, the higher rate constant was assumed to correspond to the initial reaction with O_2_. Reported rate constants are mean values with standard errors from three repeats.

##### Liquid Chromatography-Mass Spectrometry of ANR

For LC-MS, an aliquot of ANR (20 μl, 80 μm [4Fe-4S]) was combined with an equal volume of oxygenated buffer (∼220 μm O_2_) or anaerobic buffer and allowed to react for 15 min. Samples were diluted to 2.9 μm final concentration, with an aqueous mixture of 1% (v/v) acetonitrile, 0.3% (v/v) formic acid, sealed, removed from the anaerobic cabinet, and loaded (5 μl) onto a ProSwift RP-1S column (4.6 × 50 mm) (Thermo Scientific) on a Ultimate 3000 UHLPC system (Dionex, Leeds, UK). Bound protein was eluted (0.2 ml/min) using a linear gradient (15 min) from 1% to 100% (v/v) acetonitrile, 0.1% (v/v) formic acid. The eluent was continuously infused into a Bruker microQTOF-QIII mass spectrometer, running Hystar (Bruker Daltonics, Coventry, UK), using positive mode electrospray ionization. Compass Data Analysis with Maximum Entropy v1.3 (Bruker Daltonics, Coventry) was used for processing of spectra under LC peak. The mass spectrometer was calibrated with ESI-L tuning mix (Agilent Technologies).

##### Construction of Plasmids and Bacterial Strains

To investigate the responses of the three *P. putida* FNR proteins *in vivo*, it was necessary to create *P. putida* KT2440 strains that only expressed one of the three FNR proteins encoded by the genome. Two different strategies were used to create unmarked deletion mutants. The *P. putida* gene *PP_4265* encoding ANR was deleted using *sacB* counter selection and FLP recombinase excision as described by Hoang *et al.* (22). The primer pairs oAS23 (5′-GGAATTCAGCCAGATCGGCGACCTGTA-3′), oAS24 (5′-CGGGATCCTGTAGGCCAGTGTGCGCGAT-3′), oAS25 (5′-CGGGATCCACCTTGGCCTGGCGGTAGAA-3′), and oAS26 (5′-GCTCTAGACTGTCGGCATGCACTTCCAG-3′) containing engineered EcoRI, BamHI, and XbaI restrictions sites (as indicated by underlining) were used to amplify 511- and 533-bp DNA fragments flanking the *PP_4265* gene. The fragments were cloned into the suicide vector pEX18Ap flanking the gentamicin resistance cassette from plasmid pPS858 and used to generate the unmarked *P. putida PP_4265* mutant strain (22).

For the generation of unmarked gene deletion mutants of the genes encoding PP_3233 and PP_3287, the I-SceI endonuclease based knock-out strategy for *P. putida* described by Martínez-García and de Lorenzo was used (23). The following primers were used to amplify upstream and downstream regions of *PP_3233* and join both fragments by sewing PCR: *PP_3233* Upstream-Fwd (5′-GAATTCAAGGCTTTTTCGCGTTCTC-3′, engineered EcoRI site underlined), *PP_3233* Upstream-Rev (5′-GAGACCTGCATGGACGAAGGACGATGCCTCCGCTTTTTTC-3′), *PP_3233* Downstream-Fwd (5′-CTTCGTCCATGCAGGTCTC-3′), and *PP_3233* Downstream-Rev (5′-AAGCTTATTTATCGTCAGCACCCAGAGT-3′, engineered HindIII site underlined). For *PP_3287* the following primers: *PP_3287* Upstream-Fwd (5′-GAATTCTGCGATACGTAGGTAGAGCATC-3′, engineered EcoRI site underlined), *PP_3287* Upstream1-Rev (5′-AGACATCCGCAACATGAAGCTTTCAGGCCTCCTTCGCATTACG-3′), *PP_3287* Downstream-Fwd (5′-GCTTCATGTTGCGGATGTCT-3′), and *PP_3287* Downstream-Rev (5′-GGATCCCCACGTTGCATGATCTTGAG-3′, engineered BamHI site underlined) were used. The PCR products were ligated into the suicide vector pEMG and used to generate double knock-out mutants *P. putida PP_3233 PP_4265* (JRG6721) and *P. putida PP_3287 PP_4265* (JRG6722), as well as *P. putida PP_3233 PP_3287* (JRG6723) following the protocol described by Martínez-García and de Lorenzo (23).

The *PP_3233* and *PP_3287* genes including their promoter regions were amplified by PCR from *P. putida* KT2440 genomic DNA using the primer pairs PP_3233 (5′-TTTTGAATTCGGCCTGATCAACACGTGAAC-3′ and 5′-TTTTCTCGAGTCGTCAGCACCCAGAGTGC-3′) and PP_3287 (5′-TTTTGAATTCGCCAGCTACACGTTGCGAA-3′ and 5′-TTTTCTCGAGATGATCTTGAGGCGGGCGA-3′) containing engineered EcoRI and XhoI sites (underlined) for ligation into pBBR1MCS-5 to give pGS2508 and pGS2509, respectively (see Table 1).

For the heterologous reporter system, expression plasmids for use in *E. coli* JRG6348 as well as an equivalent *E. coli fnr* expression plasmid to act as a control were created (see Table 1). The open reading frames corresponding to FNR, ANR, PP_3233, and PP_3287 were amplified by PCR to incorporate a XhoI restriction site downstream of the open reading frames: MS125 (5′-ATCCCGGAAAAGCGAATTAT-3′) and MS126 (5′-TTTTCTCGAGTCAGGCAACGTTACGCGTAT-3′) for *fnr*; MS122 (5′-TCCGAGCCAGTCAAACTGCG-3′) and MS88 (5′-TTTTCTCGAGTCAGGCCTCGATTGCACCACA-3′) for *anr*; MS124 (5′-TCAGGCTCTGCAGAAATGGG-3′) and MS92 (5′-TTTTCTCGAGTCAAGTGGGCTCCTCCAGGC-3′) for *PP_3233*; MS123 (5′-CCTGGCCAGCTGAAGGTCAC-3′) and MS90 (5′-TTTTCTCGAGTCAGGGGCCCTTGGCCTCAC-3′) for *PP_3287*. After digestion with XhoI, the products were ligated into pBADHisB (Invitrogen) following NcoI and XhoI digestion and filling in the NcoI site so that the ATG start codon was provided by the vector and the encoded proteins lacked His tags. The authenticity of all constructs was confirmed by DNA sequencing.

##### In Vivo Transcription Assays

*P. putida* KT2440 mutants, JRG6721, JRG6722, and JRG6723 with deletions of two of the three FNR encoding genes, *i.e.* capable of expressing either *PP_3287*, *PP_3233*, or *anr* only, were transformed with the FNR-dependent reporter plasmid pGS810 (pFF-41.5; see Table 1). Where indicated, JRG6721 and JRG6722 were transformed with pGS810 and either pGS2508 or pGS2509 (expressing *PP_3233* or *PP_3287* under the control of their respective native promoters: see Table 1). Cultures were grown in L-broth supplemented with appropriate antibiotics—tetracycline (35 μg ml^−1^) and gentamicin (20 μg ml^−1^)—in 50 ml of shaking (200 rpm) conical flasks containing 10, 20, 30, 40, or 50 ml of medium at 30 °C for 3 h. To test the effects of nitric oxide on ANR, PP_3233, and PP_3287 activities, 1-hydroxy-2-oxo-3-(*N*-methyl-3-aminopropyl)-3-methyl-1-triazene (NOC-7, 20 μm; 40 μm nitric oxide) was added to anaerobic cultures grown in mineral medium 154 (1.4 g KH_2_PO_4_, 5.7 g Na_2_HPO_4_, 0.6 g NaCl, 1.7 g K_2_SO_4_, 0.55 mg MnSO_4_·4H_2_O, 50 mg MgSO_4_·7H_2_O, 3 mg/liter FeCl_3_) supplemented with 0.4% (w/v) yeast extract and 30 mm l-arginine and appropriate antibiotics. The effects of oxidative stress were tested in aerobic L-broth cultures supplemented with paraquat (0.2 mm). All *P. putida* cultures were incubated at 30 °C for 3 h. β-Galactosidase activities were measured as described by Miller (24).

##### RNA Isolation and qRT-PCR

Cultures of *E. coli* JRG6348 transformed with the pBAD-HisB-derivatives pGS2350, pGS2351, pGS2352, or pGS2353 (encoding FNR, PP_4265 (ANR), PP_3233, and PP_3287) all expressed under the control of the pBAD promoter to eliminate any differential transcriptional control over the production of the regulators (see Table 1) were grown under anaerobic conditions (sealed tubes filled to the neck) in M9 minimal medium supplemented with L-broth (5%, v/v), glycerol (0.4%, v/v), trimethylamine *N*-oxide (20 mm), sodium fumarate (20 mm), and ampicillin (100 μg ml^−1^) at 37 °C until the *A*_600_ reached ∼0.2 (2). Aliquots were removed, and mRNA was stabilized by the addition of 0.4 volume of ice-cold ethanol-phenol (95%:5%) at pH 4.5. The cultures were then exposed to air by shaking, and incubation was continued for 20 min before taking further samples for total RNA preparation using the RNeasy RNA purification kit (Qiagen) according to the manufacturer's instructions. RNA was quantified using a NanoDrop 1000 spectrophotometer (Thermo Scientific). Relative *lacZ* RNA quantities were determined for triplicate cultures as previously described (25).

To determine the abundances of the *anr*, *PP_3233* and *PP_3287* transcripts in *P. putida* strains, qRT-PCR was used with RNA samples isolated as described above. The genes for normalization were *gyrA* and *gyrB*. The primers used were: *anr*, 5′-TCTTTCGCTGAACCTGGAAG-3′ and 5′-AGCCAAAACTGTCACCCTG-3′; *gyrA*, 5′-GTCAACGGTTCCAGCGGTA-3′ and 5′-TTCCGGGTTGTCGATGAGC-3′; *gyrB*, 5′-GCAGCCGAGGTCATCATGA-3′ and 5′-GCGTTCACAACCGACACAC-3′; *PP_3233*, 5′-ACGAAGTGGACAAACTGGAG-3′ and 5′-GAAAATTCTTGATCGCCCCAG-3′; and *PP_3287*, 5′-GAATTTCTACCAACCTGCCATG-3′ and 5′-TTGCGGATGTCTCGTGAAG-3′.

## Results and Discussion

### 

#### 

##### P. putida

**FIGURE 1.**
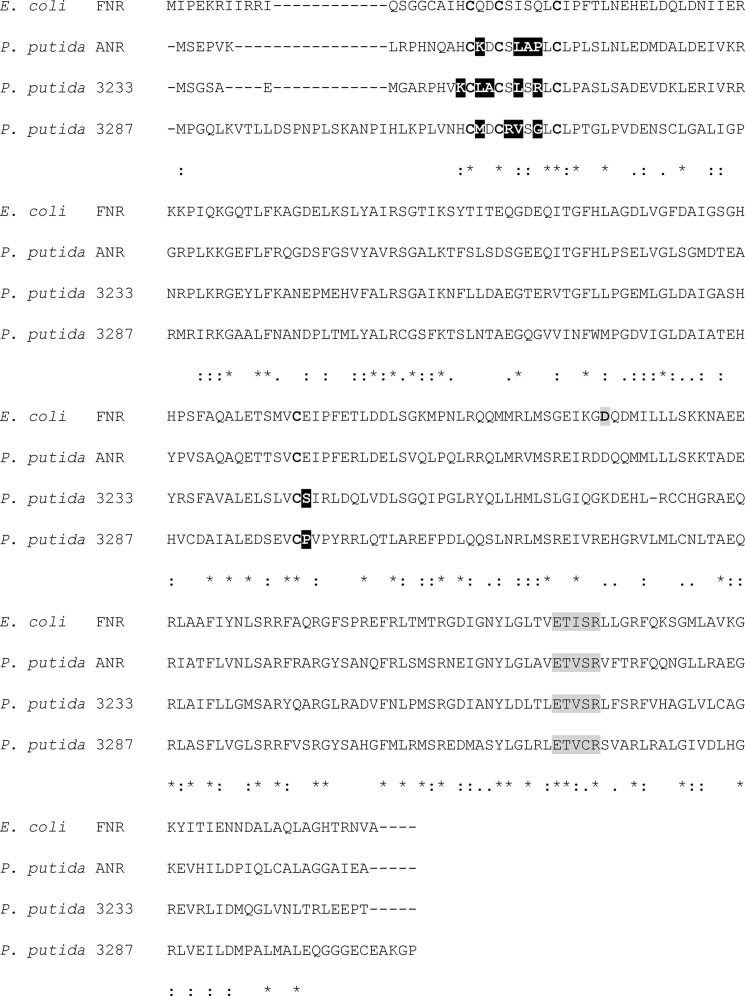
**Multiple sequence alignment of *P. putida* FNR proteins and the *E. coli* FNR protein.** Clustal Omega alignment of *E. coli* FNR and *P. putida* ANR, PP_3233, and PP_3287 proteins (41). The cysteine residues that coordinate the FNR iron-sulfur cluster (*bold type*), residues adjacent to cluster ligating cysteine residues that are substituted compared with FNR (*white type on black*), the DNA recognition helix (*shaded gray*), Asp-154 of FNR (*bold black type on gray*), residues that are identical in all four proteins (*), residues with strongly similar properties (:), and residues with weakly similar properties (.) are indicated.

**FIGURE 2.**
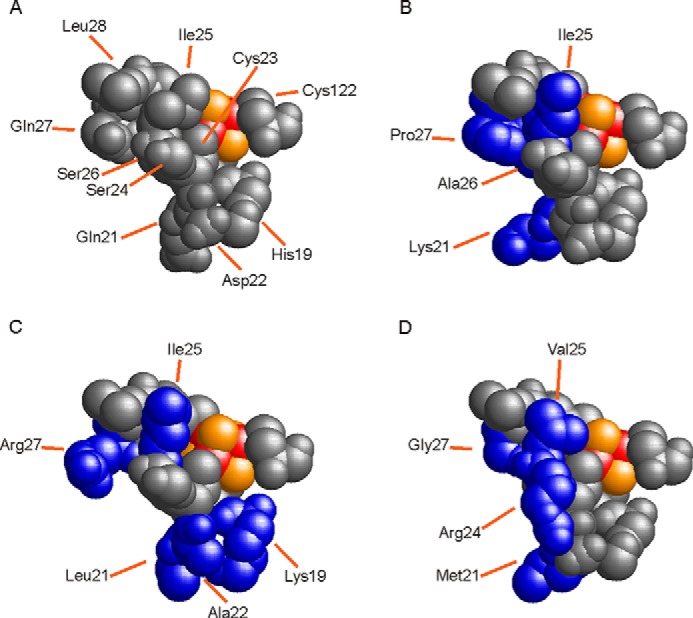
**Models of the N-terminal [4Fe-4S] cluster binding regions of four FNR proteins.** The models were constructed in SWISS-Model (42) using endonuclease III (Protein Data Bank code 2abk) as the template as described by Jervis *et al.* (12). The images were produced as space-filled representations in RasWin (43). The iron (*red*) and sulfide (*gold*) atoms of the [4Fe-4S] cluster are shown. Amino acids are labeled and numbered according to *E. coli* FNR. Conserved amino acids are colored *gray*, and those of the *P. putida* FNR proteins that differ from those present in *E. coli* FNR are colored *blue. A*, *E. coli* FNR. *B*, *P. putida* ANR. *C*, *P. putida* 3233. *D*, *P. putida* 3287.

KT2440 possesses three FNR proteins: PP_3233, PP_3287 and PP_4265 (hereafter ANR). Compared with the *E. coli* FNR protein, ANR is 53% identical (76% similar over 226 amino acid residues), PP_3233 is 46% identical (67% similar over 225 amino acid residues), and PP_3287 is 41% identical (58% similar over 224 amino acid residues). The four cysteine residues that coordinate the [4Fe-4S] cluster that is essential for the function of *E. coli* FNR are conserved, and thus all three *P. putida* FNR proteins were predicted to contain cysteine-ligated [4Fe-4S] clusters; however, the amino acid residues in the vicinity of the clusters differ (Figs. 1 and 2). Previous studies have shown that replacement of amino acid residues adjacent to cluster coordinating cysteine residues can have profound effects on the reactivity of the *E. coli* FNR iron-sulfur cluster with O_2_ (12, 26). These observations suggested that the three *P. putida* FNR proteins might have evolved different sensitivities to O_2_.

##### The Reaction of the P. putida ANR Iron-Sulfur Cluster with O_2_ Resembles That of E. coli FNR

**FIGURE 3.**
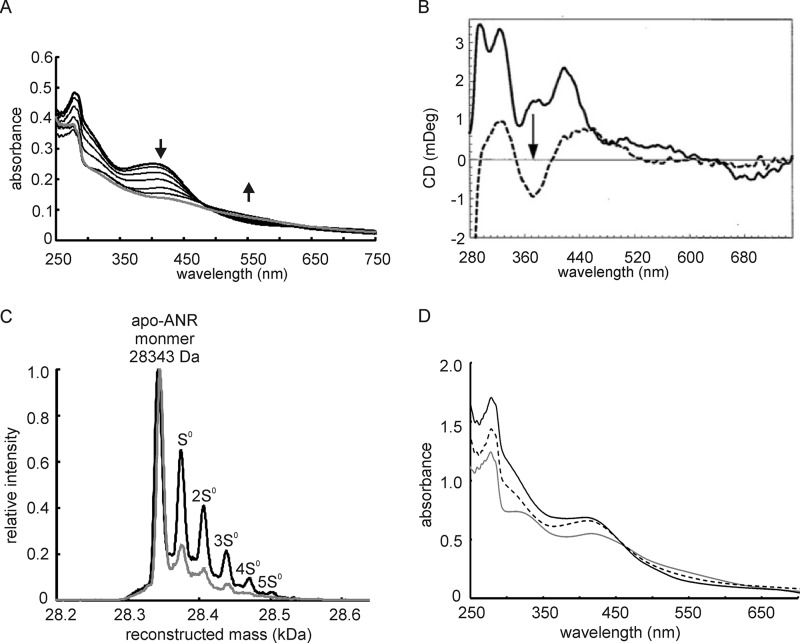
**Oxidation and repair of the *P. putida* ANR protein.** *A*, UV-visible spectrum of reconstituted ANR containing ∼15 μm [4Fe-4S] cluster under anaerobic conditions (*thick line*). The changes in the ANR spectrum upon successive additions of aerobic buffer (25 mm Tris-HCl containing 500 mm NaCl, pH 7.5) (*thin lines*) are presented along with the final spectrum ([2Fe-2S] form) shown in *gray. B*, CD spectra of [4Fe-4S] ANR (29.8 μm) before (*solid line*) and after (*dashed line*) exposure to O_2_ (∼2-fold molar excess). The *arrow* indicates the movement of spectral features in response to O_2_. The buffer was 9 mm Tris, 17 mm HEPES, 1.7 mm CaCl_2_, 236 mm NaCl, 66 mm NaNO_3_, pH 7.5. *C*, detection of persulfide forms of apo-ANR after exposure of [4Fe-4S] ANR to O_2_. Mixtures of ANR reconstituted under anaerobic conditions (initially 80 μm [4Fe-4S]^2+^ cluster) were analyzed by LC-MS after incubation with anaerobic buffer for 15 min (*gray line*) and after treatment with 2 molar equivalents of O_2_ for 15 min (*black line*). The peak at 28,343 Da corresponds to the peak ANR monomer (mass, 28,347 Da) with two disulfide bonds. The peaks labeled S^0^–5S^0^ correspond to successive S^0^ additions (+32 Da). *D*, restoration of the ANR [4Fe-4S] cluster by treatment of purified [2Fe-2S] ANR (∼40 μm cluster) with ferrous ions (4-fold molar excess) and DTT (3 mm). The *gray line* shows the initial spectrum of [2Fe-2S] ANR, the *dashed* and *solid black lines* show the spectra obtained 50 and 160 min after the addition of ferrous ions and DTT.

ANR was released from a GST-ANR fusion by “on-column” treatment with the protease thrombin. Application of the resulting apo-ANR protein to a calibrated gel filtration column indicated that unlike apo-FNR, which is monomeric (10), apo-ANR was dimeric, despite retaining Asp-154 (FNR numbering) that is proposed to cause a charge clash preventing dimerization of apo-FNR (Fig. 1 and Ref. 15). This suggests that additional residues in the dimer interface also contribute to determine the oligomeric state of ANR and FNR. After anaerobic iron-sulfur cluster reconstitution, the iron content of ANR was 4.1 ± 0.3 iron atoms per subunit (*n* = 3), based on protein estimation by total amino acid analysis. The anaerobic UV-visible spectrum of ANR was characteristic of a [4Fe-4S] protein (ϵ_405__nm_ = ∼18,000 m^−1^ cm^−1^), and upon addition of O_2_ the spectrum changed to resemble that of a [2Fe-2S] protein, with broad absorbance bands at 320, 420, and 550 nm (Fig. 3*A*). Upon prolonged (16 h) exposure to air, the [2Fe-2S] form was degraded to the apo-ANR protein. Titration of reconstituted ANR with aerobic buffer revealed a progressive decrease in absorbance in the 400–420-nm region associated with conversion of the [4Fe-4S] form to the [2Fe-2S] form (Fig. 3*A*). The CD spectrum of reconstituted [4Fe-4S] ANR exhibited positive bands at 296, 325, 375, and 420 nm, reminiscent of [4Fe-4S] FNR (21). Following exposure to O_2_ (∼2-fold molar excess), these bands were replaced by a broad spectrum with two positive bands at 325 and 450 nm and one negative band at 375 nm, similar to the [2Fe-2S] form of FNR (Fig. 3*B*) (21). Treatment of the [4Fe-4S] form of ANR with 2 molar equivalents of O_2_ for 15 min followed by analysis of the resulting [2Fe-2S] form by LC-MS revealed the presence of up to five sulfur adducts, with one and two additional sulfurs as the major species (Fig. 3*C*). Thus, it was concluded that the reaction of the ANR [4Fe-4S] cluster with O_2_ proceeds via the same mechanism as that described for FNR, including the retention of cluster sulfide (14). The retention of cluster sulfide as S^0^ has implications for the repair of [4Fe-4S] clusters (14). Anaerobic incubation of [2Fe-2S] ANR with a 4-fold molar excess of ferrous ions in the presence of the reducing agent DTT regenerated the [4Fe-4S] form, as judged by the UV-visible spectrum of the protein (Fig. 3*D*). Thus, the mechanism of [4Fe-4S] repair proposed for *E. coli* FNR is likely to be a common feature of this family of regulators and probably other iron-sulfur proteins (14).

##### Reactions of the [4Fe-4S] Clusters of PP_3233 and PP_3287 with O_2_ Result in Conversion to [2Fe-2S] Forms

Several attempts were made to overproduce the *P. putida* PP_3233 and PP_3287 proteins, but they were consistently found as insoluble aggregates when expressed at high levels, except when fused to the C terminus of the chaperone Trigger factor (Tig). Therefore, PP_3233 and PP_3287 were isolated as Tig fusions, and a Tig fusion of ANR was also generated to permit direct comparisons.

**FIGURE 4.**
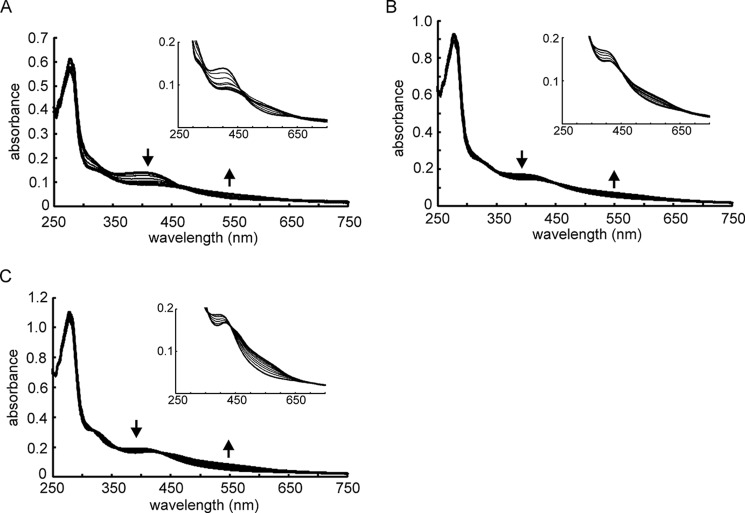
**Absorbance spectra of Tig-tagged *P. putida* FNR proteins after treatment with increasing amounts of O_2_.** Absorbance spectra obtained by titration of anaerobic solutions of proteins with air-saturated (220 μm O_2_ at 25 °C) buffer (25 mm Tris-HCl containing 500 mm NaCl, pH 7.5). The initial and final spectra are shown as *bold lines*. After each O_2_ addition, the sample was incubated for 10 min at 25 °C before obtaining the spectrum shown. The *arrows* indicate the direction of spectral change during the titration. *A*, ANR. *B*, PP_3233. *C*, PP_3287. The *insets* show the spectral changes in the visible region with an expanded ordinate (absorbance) scale. The spectra shown are typical of at least three measurements.

Anaerobic reconstitution of the iron-sulfur clusters of the three Tig fusion proteins resulted in UV-visible spectra characteristic of [4Fe-4S] proteins, with a broad absorbance at 400–420 nm (Fig. 4, *A–C*). Titration with aerobic buffer resulted in spectral changes that were consistent with conversion from [4Fe-4S] to [2Fe-2S] forms (Fig. 4, *A–C*). The response of the Tig-ANR fusion (Fig. 4*A*) was similar to that of the untagged ANR protein (Fig. 3*A*), suggesting that the Tig tag did not impair cluster acquisition or O_2_-mediated cluster conversion. Thus, it was concluded that all the *P. putida* FNR proteins acquired [4Fe-4S] clusters that underwent conversion to [2Fe-2S] clusters in the presence of O_2_.

##### PP_3233 and PP_3287 React More Slowly than ANR with O_2_ in Vitro

**FIGURE 5.**
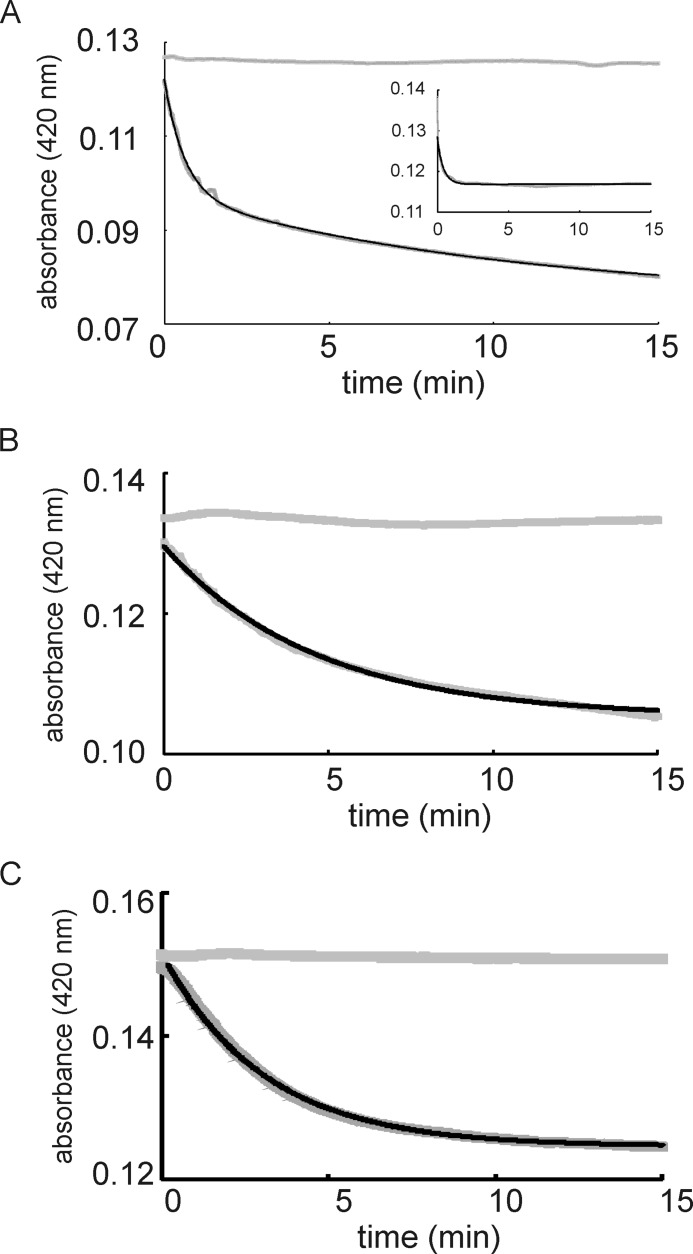
**Kinetics of O_2_-mediated [4Fe-4S] cluster conversion.** Samples of reconstituted Tig fusions of ANR (*A*, *inset* untagged ANR), PP_3233 (*B*), and PP_3287 containing ∼8 μm [4Fe-4S] cluster (*C*) were mixed with a 13-fold molar excess of O_2_ as aerobic buffer at 25 °C. The buffer was 25 mm HEPES, 100 mm NaCl, 100 mm NaNO_3_, pH 7.5. Loss of the [4Fe-4S] clusters was monitored at 420 nm as a function of time (*gray lines*). Data were fitted to exponential functions as described in the text (*black lines*). The *upper data set* (not fitted) in each panel shows the response when anaerobic buffer was used in place of aerobic buffer. The rate constants reported in the text from these experiments are mean values with standard errors from three repeats.

Under pseudo-first order reaction conditions (O_2_:[4Fe-4S] ratio of ∼13), the *A*_420 nm_ decays for ANR (both ANR and the Tig-ANR fusion), Tig-PP_3233, and Tig-PP_3287 were measured (Fig. 5). For ANR and Tig-ANR, the data were best fitted to a double-exponential function with observed rate constants (*k*_obs_) for the first reaction of 0.034 ± 0.007 s^−1^ for ANR and 0.028 ± 0.0015 s^−1^ for the Tig-ANR fusion (Fig. 5*A*). This again indicates that fusion to Tig did not significantly affect the reactivity of the ANR iron-sulfur cluster, and thus it was assumed that a Tig tag would not affect the reactivity of the PP_3233 and PP_3287 clusters. For the Tig-PP_3233 and Tig-PP_3287 fusion proteins, the data were best fitted to a single-exponential function yielding *k*_obs_ values of 0.0038 ± 0.0002 s^−1^ for Tig-PP_3233 and 0.0055 ± 0.0001 s^−1^ for Tig-PP_3287 (Fig. 5, *B* and *C*). Division of the observed rate constants by the O_2_ concentration provides an estimate of the apparent second order rate constants for the fusion proteins: ANR, 280 m^−1^ s^−1^ (*cf*. 309 m^−1^ s^−1^ for the untagged ANR); PP_3233, 38 m^−1^ s^−1^; and PP_3287, 55 m^−1^ s^−1^. These values indicate that the [4Fe-4S] cluster of ANR displays similar sensitivity to O_2_ as previously reported for *E. coli* FNR (278 m^−1^ s^−1^), but the iron-sulfur clusters of PP_3233 and PP_3287 were significantly less reactive with O_2_ *in vitro*, more closely resembling the previously characterized variant FNR-S24F (80 m^−1^ s^−1^), which is also less responsive to O_2_ *in vivo* (11, 12).

##### The Responses of PP_3233 and PP_3287 to Increased Culture Aeration Are Weaker than That of ANR

**FIGURE 6.**
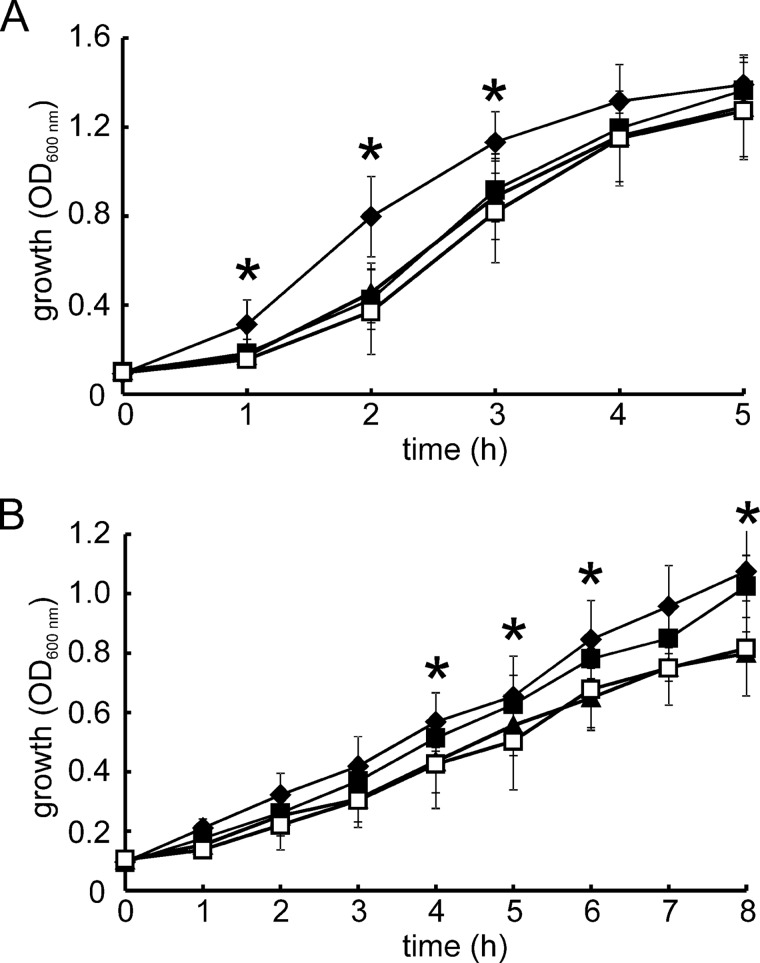
**Growth of *P. putida* under aerobic and O_2_-limited conditions.** *P. putida* mutants that lack two of the three *fnr* genes present in the wild-type strain were grown under aerobic conditions (*A*, 10 ml of medium in a 50-ml conical flask with shaking at 200 rpm, 30 °C) or O_2_-limiting conditions (*B*, 40 ml of medium in a 50-ml conical flask with shaking at 200 rpm, 30 °C). Growth was monitored by measuring the optical density of the cultures (*A*_600 nm_). Wild-type (ANR^+^, PP_3233^+^, PP_3287^+^; *filled diamonds*); JRG6723 (ANR^+^; *filled squares*); JRG6722 (PP_3233^+^; *filled triangles*); JRG6721 (PP_3287^+^; *open squares*). The data points show the means and standard deviations (*n* = 6). *, significant difference between: wild-type (*A*) and all mutant strains and strains possessing the *anr* gene (*i.e.* wild-type and JRG6723) and those lacking *anr* (JRG6721 and JRG6722) (*p* ≤ 0.05) (*B*).

To determine whether ANR, PP_3233, and PP_3287 act as O_2_ sensors *in vivo*, three double mutant strains of *P. putida* were created in which two of the three genes encoding FNR proteins were deleted (Table 1). Cultures of these strains were grown in 50-ml conical flasks at 30 °C with shaking (200 rpm). For aerobic cultures, the flasks contained 10 ml of medium; for O_2_-limited cultures, the flasks contained 40 ml of medium. Strains that lacked *anr* exhibited impaired growth under O_2_-limited conditions, and strains that lacked either *PP_3233* or *PP_3287* were impaired under aerobic conditions (Fig. 6). This is consistent with relative O_2_ sensitivities of the ANR, PP_3233, and PP_3287 iron-sulfur clusters.

**TABLE 1:** **Bacterial strains and plasmids used in this work** Amp^R^, ampicillin resistance; Cm^R^, chloramphenicol resistance; Gm^R^, gentamicin resistance; Kan^R^, kanamycin resistance; Tet^R^, tetracycline resistance.

	Relevant characteristics	Source
**Bacterial strain**		
JRG6348	A derivative of *E. coli* JRG1728 *lac*, *fnr* carrying a chromosomal FNR-dependent promoter-*lacZ* fusion; Cm^R^	Dr. David Lee (University of Birmingham, Birmingham, UK)
JRG6725	*P. putida* KT2440 parent strain; source of genomic DNA for amplification and cloning *anr*, *PP_3233*, and *PP_3287* genes for protein expression and construction of JRG6721, JRG6722, and JRG6723	Laboratory collection
JRG6721	*P. putida* KT2440 *PP_3233*, *anr* (PP_3287^+^)	This work
JRG6722	*P. putida* KT2440 *PP_3287*, *anr* (PP_3233^+^)	This work

JRG6723	*P. putida* KT2440 *PP_3233, PP_3287* (ANR^+^)	This work
**Plasmid**		
pAS12	pEX18Ap containing gene replacement cassette for *PP_4265*, Amp^R^, Gm^R^	This study
pBBR1-MCS-5	Broad host range vector, Kan^R^.	Ref. 35
pDelta_PP_3233	pEMG containing replacement cassette for PP3233, Kan^R^	This study
pDelta_PP_3287	pEMG containing replacement cassette for PP3287, Kan^R^	This study
pEMG	Gene replacement vector with two flanking I-SceI sites, Kan^R^	Ref. 23
pEX18Ap	Gene replacement vector, *sacB*, Amp^R^	Ref. 22
pFLP2	Broad host range vector with FLP recombinase, Amp^R^	Ref. 22
pGS422	pUC13 containing the FF-41.5 promoter (EcoRI-HindIII)	Ref. 36
pGS652	pBluescript containing the NN-41.5 promoter (EcoRI-HindIII)	Ref. 37
pGS810 (pFF-41.5)	pRW50 derivative with *lacZ* under the control of a class II FNR-dependent promoter, Tet^R^	Ref. 38
pGS2268	pGEX-KG (39) containing ANR for expression as a GST fusion protein; Amp^R^	This work
pGS2350	pBAD-HisB (Invitrogen) derivative for expression of *E. coli fnr*, Amp^R^	This work
pGS2351	pBAD-HisB derivative for expression of *P. putida anr*; Amp^R^	This work
pGS2352	pBAD-HisB derivative for expression of *P. putida PP_3233*, Amp^R^	This work
pGS2353	pBAD-HisB derivative for expression of *P. putida PP_3287*, Amp^R^	This work
pGS2403	As for pGS2414 but containing *PP_3233*	This work
pGS2413	As for pGS2414 but containing *PP_3287*	This work
pGS2414	pCOLD-TF (Takara Clontech) containing *anr* for expression as a His_6_-Tig fusion protein, Amp^R^	This work
pGS2508	*PP_3233* ligated into pBB1MSC-5 (EcoRI-XhoI) for expression of *PP_3233* in *P. putida*, Gm^R^	This work
pGS2509	*PP_3287* ligated into pBB1MSC-5 (EcoRI-XhoI) for expression of *PP_3287* in *P. putida*; Gm^R^	This work
pPS858	Source of gentamicin resistance cassette; Amp^R^, Gm^R^	Ref. 22
pSWI	Broad host range vector with I-SceI endonuclease, Amp^R^	Ref. 40

**FIGURE 7.**
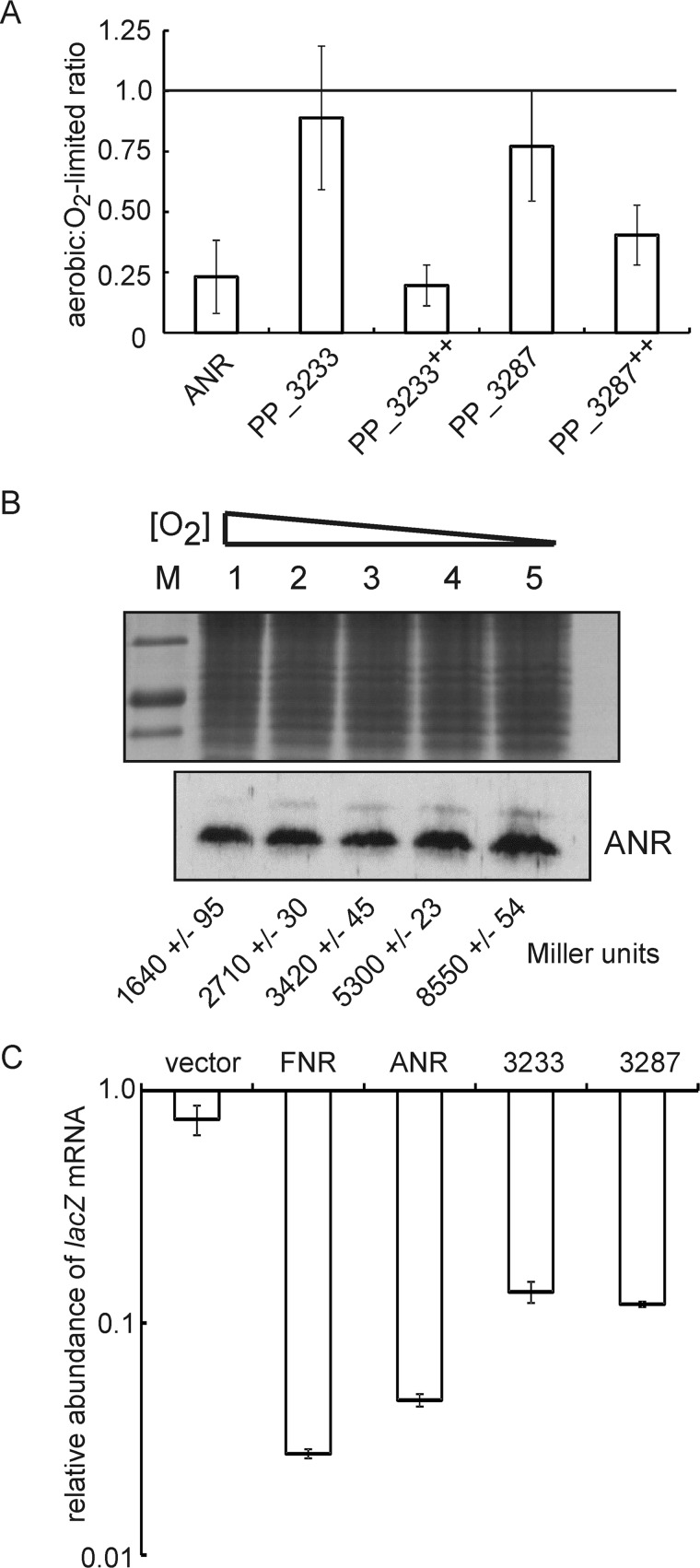
**Responses of *P. putida* FNR proteins to O_2_*in vivo*.** *A*, the output from an FNR-dependent promoter decreases in response to enhanced aeration of *P. putida* cultures expressing only one of the three FNR proteins. All the strains were transformed with the FF-41.5-*lacZ* reporter plasmid pGS810. The rate of culture aeration was increased by decreasing the volume of medium in the shaking conical flasks (50 ml of medium for O_2_-limited cultures and 10 ml of medium for aerobic cultures). Cultures were grown at 30 °C for 3 h, at which point samples were taken for measurement of β-galactosidase activity. The β-galactosidase activities of the aerobic cultures were divided by those of the O_2_-limited cultures. The *error bars* are the standard deviation from the mean values of the aerobic:O_2_-limited ratios (*n* = 4). *ANR*, *PP_3233*, and *PP_3287* indicate chromosomal expression of the corresponding genes; *PP_3233*^++^ and *PP_3287*^++^ indicate expression of the corresponding genes from a multicopy plasmid. *B*, concentration of cytoplasmic ANR does not respond to changes in culture aeration. Shown are Western blots developed with anti-serum raised against *E. coli* FNR for cell samples from *P. putida* cultures that express only *anr* grown in shaking 50-ml conical flasks containing 10, 20, 30, 40, or 50 ml of medium (*lanes 1–5*) to impose an increasing degree of O_2_ limitation on the cultures. The equivalent region of a Coomassie Blue-stained gel is shown as a loading control (*M* indicates protein standard markers: 37, 25, and 20 kDa, *top* to *bottom*). The outputs from the pFF-41.5 reporter (pGS810) for cultures grown as described above are shown below each lane (mean values ± standard deviation, *n* = 3). *C*, inactivation of FNR proteins upon exposure of anaerobic cultures to air. Cultures of *E. coli* JRG6348 expressing either no FNR (vector), *E. coli* FNR, *P. putida* ANR, PP_3233, or PP_3287, as indicated, were grown under anaerobic conditions and the abundance of FNR-protein-dependent *lacZ* transcription was measured by qRT-PCR. The cultures were exposed to air for 20 min, and then the abundance of the *lacZ* transcript was measured again. The relative abundance of *lacZ* mRNA after transfer to aerobic conditions is shown. The *error bars* are the standard deviation from the mean (*n* = 3).

For *E. coli* FNR, it has been shown that Glu-209, Ser-212, and Arg-213 in the DNA recognition helix make the major interactions with the FNR box: TTGATCTAGATCAA (FF site). The amino acid sequences of the DNA recognition helices of the *P. putida* FNR proteins are very similar to those of *E. coli* FNR (PP_3287 has Cys in place of Ser), suggesting that ANR, PP_3233, and PP_3287 will recognize an FNR box (Fig. 1). Preliminary electromobility shift assays indicated that all three *P. putida* FNR proteins could bind at the FF site under anaerobic conditions. Therefore, the *P. putida* mutants were transformed with plasmid pGS810, which carries the FNR-dependent FF-41.5 (pFF-41.5) promoter fused to *lacZ* (Table 1). Cultures were grown under O_2_-limited conditions (50 ml of medium in a 50-ml shaking conical flask) and aerobic conditions (10 ml of medium in a 50-ml shaking conical flask). Measurement of β-galactosidase activity for *P. putida* JRG6723 (expresses only *anr*) cultures showed that ANR activity decreased ∼5-fold in response to enhanced aeration (Fig. 7, *A* and *B*). Measurement of the *anr* transcript by qRT-PCR and ANR protein by Western blotting with anti-serum raised against *E. coli* FNR for cultures grown in 50-ml shake flasks containing 50, 40, 30, 20, or 10 ml of medium, resulting in increasing O_2_ transfer to the cultures as the volume of medium decreased, showed that the amounts of *anr* transcript and ANR protein were similar in all the cultures (Fig. 7*B*). However, measurement of ANR-dependent transcription in cultures grown under these same conditions showed that increased aeration progressively lowered FF-41.5 promoter activity (Fig. 7*B*). Thus, it was concluded that ANR has properties similar to *E. coli* FNR and is a *bona fide* O_2_-responsive gene regulator in *P. putida*. However, β-galactosidase activities for the *P. putida* strains lacking the *anr* gene (*i.e. P. putida* JRG6722 expresses only *PP_3233*, and *P. putida* JRG6721 expresses only *PP_3287*) showed only a small decrease in response to increased aeration and low levels of β-galactosidase activity (128 ± 6 and 184 ± 3 Miller units, respectively, under O_2_-limited conditions compared with 8550 ± 54 Miller units for ANR) (Fig. 7*A*). This observation could result from poor expression of the PP_3233 and PP_3287 proteins. Therefore qRT-PCR was used to determine whether *PP_3233* and *PP_3287* were expressed in JRG6722 and JRG6721 under the conditions tested. The data indicated that the level of expression of *PP_3233* was ∼10-fold lower, and *PP_3287* was ∼5-fold lower than *anr*. Low levels of *PP_3233* and *PP_3287* mRNA were consistent with the hypothesis that expression of the three *P. putida* FNR proteins is likely to be temporally and/or spatially distinct. Therefore, to increase expression of *PP_3233* and *PP_3287*, these genes and their respective promoter regions were ligated into the broad host range vector pBBR1-MCS-5 (Table 1). The resulting expression plasmids were used to transform *P. putida* JRG6722 and JRG6721 carrying the pGS810 reporter plasmid creating strains that only expressed PP_3233 or PP_3287. Cultures expressing *PP_3233* from pGS2508 (ANR^−^, PP_3233^++^, PP_3287^−^) or *PP_3287* from pGS2509 (ANR^−^, PP_3233^−^, PP_3287^++^) were grown under O_2_-limited and aerobic conditions, and qRT-PCR showed that the level of *PP_3233* and *PP_3287* expression was increased by ∼10- and ∼5-fold compared with the expression of chromosomal *anr*. Unfortunately, the corresponding increase in PP_3233 and PP_3287 proteins could not be determined because the *E. coli* FNR anti-serum did not cross-react with these proteins. Nevertheless, for both overexpression strains, FNR-dependent β-galactosidase activitydecreased significantly with increased aeration (Fig. 7*A*). Thus, it was concluded that transcription activation by PP_3233 and PP_3287 was inhibited by O_2_.

To confirm the *in vivo* O_2_ responsiveness of the *P. putida* FNR proteins, a heterologous reporter system consisting of an *E. coli fnr, lac* mutant (JRG6348) with a chromosomal copy of the FNR-dependent FF-41.5 promoter fused to *lacZ* was transformed with plasmids expressing FNR, ANR, PP_3233, or PP_3287 under the control of the pBAD promoter (Table 1). Measurement of the decrease in *lacZ* transcript abundance by qRT-PCR after exposure of anaerobic cultures to O_2_ for 20 min showed that the activities of *E. coli* FNR and all three *P. putida* regulators decreased, with FNR- and ANR-dependent transcription showing the greatest responses (Fig. 7*C*). The weaker responses of PP_3233 and PP_3287 suggested that these proteins were less sensitive to O_2_ compared with FNR and ANR, consistent with the *in vitro* data presented above.

##### Signal Specificity

**FIGURE 8.**
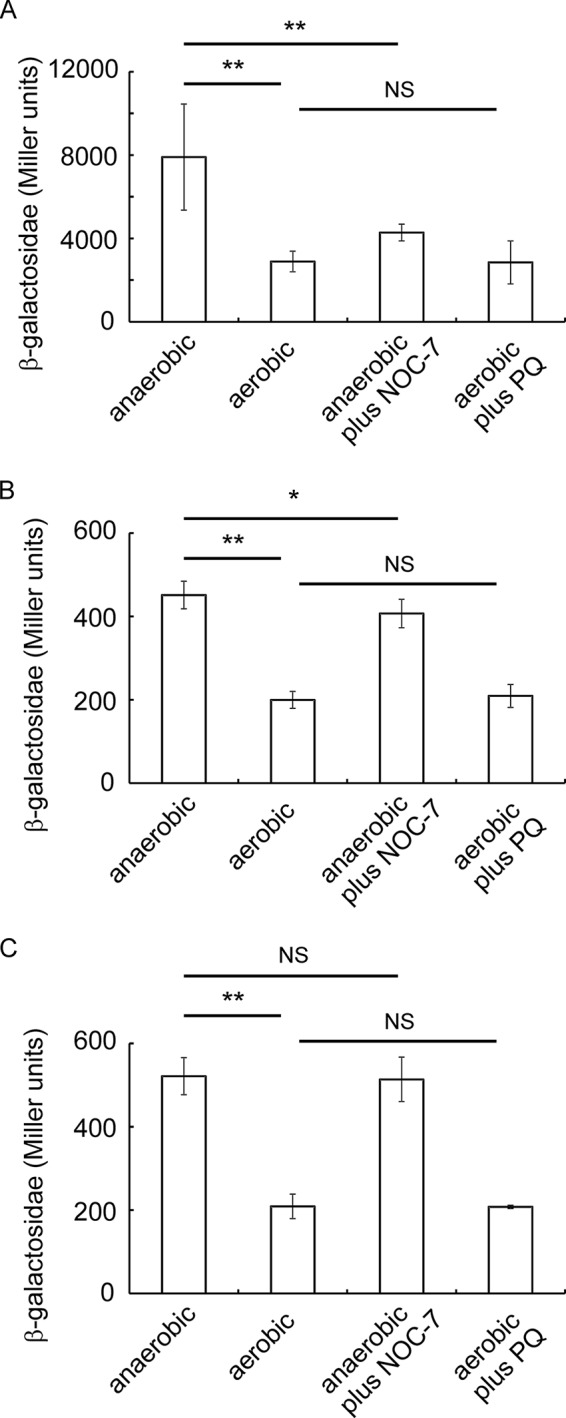
**Responses of *P. putida* FNR proteins to nitric oxide and oxidative stress *in vivo*.** *P. putida* strains capable of expressing only *anr* from the chromosome (ANR) or only *PP_3233* from a multicopy plasmid (PP_3233^++^) or only *PP_3287* from a plasmid (PP_3287^++^) were transformed with the FF-41.5-*lacZ* reporter plasmid pGS810. Cultures were grown at 30 °C for 3 h under anaerobic (50 ml of anaerobic minimal medium 154 supplemented with 0.4% (w/v) yeast extract and 30 mm l-arginine in a sealed 50-ml conical flask) or aerobic (10 ml of L-broth in a 50-ml conical flask, 200 rpm shaking) conditions in the presence and absence of NOC-7 (20 μm) or paraquat (*PQ*; 200 μm) as indicated. Samples were taken for measurement of β-galactosidase activity as a proxy for FNR protein activity: ANR (*A*), PP_3233^++^ (*B*), and PP_3287^++^ (*C*). The *error bars* are the standard deviation from the mean values (*n* = 3).). *p* values were determined by Student's *t* test. **, *p* < 0.01; *, *p* < 0.1; *NS*, *p* > 0.5.

Transcription factors that utilize iron-sulfur clusters as sensory modules have been shown to respond to O_2_ (*e.g.* FNR), redox state (*e.g.* SoxR), nitric oxide (*e.g.* NsrR), and iron-sulfur cluster/iron homeostasis (*e.g.* IscR) (27, 28). Some of these transcription factors respond to more than one of these signals. Hence, the *E. coli* FNR and SoxR proteins respond to nitric oxide in addition to their primary signals, O_2_ and redox cycling, respectively. *In vitro* kinetic measurements with the [4Fe-4S] form of FNR indicated that it is much more sensitive to nitric oxide than it is to O_2_. However, *in vivo*, FNR is only nitrosylated when the major nitric oxide sensors (*e.g.* NsrR and NorR) and detoxification systems (*e.g.* NorVW, NrfA, and Hmp) are overwhelmed. Thus, FNR serves primarily as an O_2_ sensor with a secondary nitric oxide sensing role (25). By contrast, the iron-sulfur clusters of regulators that are primarily nitric oxide sensors (*e.g.* NsrR and Wbl proteins) or redox sensors (*e.g.* SoxR) are generally stable for several hours in the presence of O_2_ (29–31). The data described above show that the three *P. putida* FNR proteins respond to O_2_ *in vitro* and *in vivo*, suggesting that they are primarily O_2_ sensors. To determine whether they also share the nitric oxide- or redox-responsive characteristics of *E. coli* FNR and SoxR, respectively, anaerobic cultures of *P. putida* expressing only one of the three FNR proteins and carrying the FNR-dependent pFF-41.5 fused to *lacZ* were supplemented with the nitric oxide donor NOC-7; in addition, aerobic cultures were exposed to the redox cycling agent paraquat. The responses of PP_3233 and PP_3287 were similar, nitric oxide had little or no effect under anaerobic conditions, and paraquat had no effect under aerobic conditions (Fig. 8). However, for ANR, nitric oxide significantly inactivated anaerobic reporter gene expression, whereas paraquat again had no effect under aerobic conditions (Fig. 8). Thus, the response of ANR was similar to that reported previously for *E. coli* FNR, further confirming the similarities between these two proteins, but PP_3233 and PP_3287 were less responsive with both O_2_ and nitric oxide compared with ANR (25). Nevertheless, in all cases the greatest responses were provoked by culture aeration, and therefore, it was concluded that O_2_ is the major modulator of the activity of all three *P. putida* FNR proteins.

##### Conclusions

The research described here suggests that the three FNR proteins of *P. putida* have evolved to fulfill distinct but overlapping roles. All three regulators, ANR, PP_3233, and PP_3287, acquired [4Fe-4S] clusters under anaerobic conditions and were converted to [2Fe-2S] forms upon exposure to O_2_ *in vitro*. ANR has the least number of nonconservative amino acid substitutions in the vicinity of the cluster-ligating cysteine residues compared with *E. coli* FNR and hence was expected to exhibit similar cluster reactivity to FNR (Fig. 1). The double-exponential nature of the ANR [4Fe-4S] cluster reaction with O_2_, the detection of sulfur adducts after conversion of [4Fe-4S]^2+^ ANR to the [2Fe-2S] form, and the capacity to repair the [4Fe-4S] cluster by simply providing ferrous ions under reducing conditions are consistent with the reaction scheme for *E. coli* FNR and O_2_ proposed by Zhang *et al.* (14) in which the [2Fe-2S]^2+^ cluster is ligated by one (Equations 5 and 6) or two (Equations 5 and 7) cysteine persulfides (CysSS).

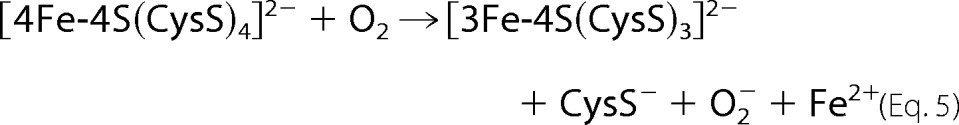


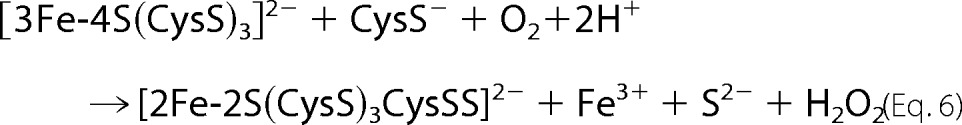


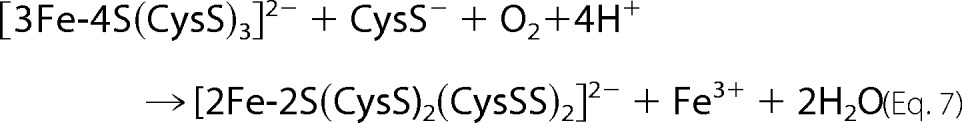
 Furthermore, ANR resembled FNR in exhibiting a secondary response when cultures were exposed to micromolar levels of nitric oxide (Fig. 8). Thus, the observations reported here are consistent with *P. putida* ANR acting as an *E. coli*-type O_2_ sensor regulator, in accordance with its ability to regulate the expression of multiple terminal oxidases of the *P. putida* respiratory chain and the ability of the closely related *P. aeruginosa anr* gene (encoded protein 88% identical, 94% similar over 244 amino acids) to complement the anaerobic growth phenotype of an *E. coli fnr* mutant (32, 33).

The [4Fe-4S] clusters of PP_3233 and PP_3287 also underwent conversion to [2Fe-2S] clusters upon exposure to O_2_, but these reactions were slower than that of ANR, and the responses of these proteins when cultures were exposed to nitric oxide were weaker than that observed for ANR (Fig. 8). The kinetic data for the reaction of the PP_3233 and PP_3287 [4Fe-4S] clusters with O_2_ fitted well to a single-exponential function rather than a double-exponential function, implying that, unlike FNR and ANR, the initial cluster oxidation step to generate the [3Fe-4S]^1+^ intermediate (Equation 5) was much slower than the subsequent decay of the [3Fe-4S]^1+^ to the [2Fe-2S]^2+^ form (Equations 6 and 7). Thus, it is suggested that the mechanism for [4Fe-4S] to [2Fe-2S] cluster conversion in PP_3233 and PP_3287 was similar to that described for FNR (11) and ANR, but the PP_3233 and PP_3287 [4Fe-4S] clusters appear to be more stable when bacteria are exposed to air or nitric oxide. The relative rates of ANR, PP_3233, and PP_3287 cluster reactions with O_2_ results in differential responses to O_2_ availability.

The *in vivo* properties of ANR, PP_3233, and PP_3287 were consistent with the observed reactivities of the [4Fe-4S] clusters with O_2_. Previous work with *E. coli* FNR showed that replacement of Ser-24, which is located immediately adjacent to the cluster ligand Cys-23, by Arg resulted in significant aerobic FNR activity, indicative of stabilization of the FNR-S24R iron-sulfur cluster (12). Interestingly, PP_3287 has Arg in the position equivalent to Ser-24 in FNR (Figs. 1 and 2), and thus this amino acid substitution could at least partially account for the lower reactivity of PP_3287 with O_2_. On the other hand, PP_3233 resembles *E. coli* FNR by retaining a Ser residue at the equivalent of position 24 (Figs. 1 and 2); however, amino acid substitutions in other locations are known to influence the reactivity of the *E. coli* FNR iron-sulfur cluster with O_2_ (26, 34). Like S24R, another amino acid substitution that promoted aerobic FNR activity was also located immediately adjacent to Cys-23, but this time on the other flank (D22G) (34). The equivalent position in PP_3233 is occupied by Ala (Figs. 1 and 2), and thus by analogy, replacement of the acidic Asp residue might alter the redox properties of the PP_3233 iron-sulfur cluster, such that it is less O_2_ reactive.

Although the observations reported here resolve several aspects of the properties of the three FNR proteins possessed by *P. putida* KT2440, many questions remain, including: (i) What are the conditions encountered by *P. putida* that induce *PP_3233* and *PP_3287* target gene expression? (ii) Do the three *P. putida* FNR proteins control distinct but overlapping regulons, perhaps by making productive interactions with additional transcription factors or alternative sigma factors? (iii) What is the imperative for employing multiple FNR proteins to extend the range of O_2_-responsive gene expression? Further detailed biochemical and physiological studies are now required to address these questions and in so doing discern the mechanism of the observed differential sensitivities to O_2_ of these closely related proteins and the broader implications for the control of gene expression in *P. putida*.

## Footnotes

2 The abbreviations used are:

FNR: fumarate-nitrate reduction regulator
ANR: arginine deiminase nitrate reductase regulator
NOC-7: 1-hydroxy-2-oxo-3-(*N*-methyl-3-aminopropyl)-3-methyl-1-triazene
qRT-PCR: quantitative RT-PCR.

## References

[1] Körner H. Sofia H. J. Zumft W. G. (2003) Phylogeny of the bacterial superfamily of Crp-Fnr transcription regulators: exploiting the metabolic spectrum by controlling alternative gene programs. FEMS Microbiol. Rev. 27, 559–592 14638413 10.1016/S0168-6445(03)00066-4

[2] Constantinidou C. Hobman J. L. Griffiths L. Patel M. D. Penn C. W. Cole J. A. Overton T. W. (2006) A reassessment of the FNR regulon and transcriptomic analysis of the effects of nitrate, nitrite, NarXL, and NarQP as *Escherichia coli* K12 adapts from aerobic to anaerobic growth. J. Biol. Chem. 281, 4802–4815 16377617 10.1074/jbc.M512312200

[3] Kang Y. Weber K. D. Qiu Y. Kiley P. J. Blattner F. R. (2005) Genome-wide expression analysis indicates that FNR of *Escherichia coli* K-12 regulates a large number of genes of unknown function. J. Bacteriol. 187, 1135–1160 15659690 10.1128/JB.187.3.1135-1160.2005 PMC545700

[4] Myers K. S. Yan H. Ong I. M. Chung D. Liang K. Tran F. Keleş S. Landick R. Kiley P. J. (2013) Genome-scale analysis of *Escherichia coli* FNR reveals complex features of transcription factor binding. PLoS Genet. 9, e1003565 23818864 10.1371/journal.pgen.1003565 PMC3688515

[5] Partridge J. D. Scott C. Tang Y. Poole R. K. Green J. (2006) *Escherichia coli* transcriptome dynamics during transition from anaerobic to aerobic conditions. J. Biol. Chem. 281, 27806–27815 16857675 10.1074/jbc.M603450200

[6] Partridge J. D. Sanguinetti G. Dibden D. P. Roberts R. E. Poole R. K. Green J. (2007) Transition of *Escherichia coli* from aerobic to micro-aerobic conditions involves fast and slow reacting components. J. Biol. Chem. 282, 11230–11237 17307737 10.1074/jbc.M700728200

[7] Rolfe M. D. Ocone A. Stapleton M. R. Hall S. Trotter E. W. Poole R. K. Sanguinetti G. Green J. (2012) Systems analysis of transcription factor activities in environments with stable and dynamic oxygen concentrations. Open Biol. 2, 120091 22870390 10.1098/rsob.120091 PMC3411108

[8] Sharrocks A. D. Green J. Guest J. R. (1990) *In vivo* and *in vitro* mutants of FNR the anaerobic transcriptional regulator of *E. coli*. FEBS Lett. 270, 119–122 2226775 10.1016/0014-5793(90)81248-m

[9] Khoroshilova N. Popescu C. Münck E. Beinert H. Kiley P. J. (1997) Iron-sulfur cluster disassembly in the FNR protein of *Escherichia coli* by O_2_: [4Fe-4S] to [2Fe-2S] conversion with loss of biological activity. Proc. Natl. Acad. Sci. U.S.A. 94, 6087–6092 9177174 10.1073/pnas.94.12.6087 PMC21006

[10] Lazazzera B. A. Beinert H. Khoroshilova N. Kennedy M. C. Kiley P. J. (1996) DNA binding and dimerization of the Fe-S-containing FNR protein from *Escherichia coli* are regulated by oxygen. J. Biol. Chem. 271, 2762–2768 8576252 10.1074/jbc.271.5.2762

[11] Crack J. C. Green J. Cheesman M. R. Le Brun N. E. Thomson A. J. (2007) Superoxide-mediated amplification of the oxygen-induced switch from [4Fe-4S] to [2Fe-2S] clusters in the transcriptional regulator FNR. Proc. Natl. Acad. Sci. U.S.A. 104, 2092–2097 17267605 10.1073/pnas.0609514104 PMC1892919

[12] Jervis A. J. Crack J. C. White G. Artymiuk P. J. Cheesman M. R. Thomson A. J. Le Brun N. E. Green J. (2009) The O_2_ sensitivity of the transcription factor FNR is controlled by Ser24 modulating the kinetics of the [4Fe-4S] to [2Fe-2S] cluster conversion. Proc. Natl. Acad. Sci. U.S.A. 106, 4659–4664 19261852 10.1073/pnas.0804943106 PMC2660729

[13] Reinhart F. Achebach S. Koch T. Unden G. (2008) Reduced apo-fumarate nitrate reductase regulator (apoFNR) as the major form of FNR in aerobically growing *Escherichia coli*. J. Bacteriol. 190, 879–886 18055593 10.1128/JB.01374-07 PMC2223584

[14] Zhang B. Crack J. C. Subramanian S. Green J. Thomson A. J. Le Brun N. E. Johnson M. K. (2012) Reversible cycling between cysteine persulfide-ligated [2Fe-2S] and cysteine-ligated [4Fe-4S] clusters in the FNR regulatory protein. Proc. Natl. Acad. Sci. U.S.A. 109, 15734–15739 23019358 10.1073/pnas.1208787109 PMC3465412

[15] Moore L. J. Mettert E. L. Kiley P. J. (2006) Regulation of FNR dimerization by subunit charge repulsion. J. Biol. Chem. 281, 33268–33275 16959764 10.1074/jbc.M608331200

[16] Dibden D. P. Green J. (2005) *In vivo* cycling of the *Escherichia coli* transcription factor FNR between active and inactive states. Microbiology 151, 4063–4070 16339951 10.1099/mic.0.28253-0

[17] Engel P. Trageser M. Unden G. (1991) Reversible interconversion of the functional state of the gene regulator FNR from *Escherichia coli in vivo* by O_2_ and iron availability. Arch. Microbiol. 156, 463–470 1785953 10.1007/BF00245393

[18] Mettert E. L. Kiley P. J. (2005) ClpXP-dependent proteolysis of FNR upon loss of its O_2_-sensing [4Fe-4S] cluster. J. Mol. Biol. 354, 220–232 16243354 10.1016/j.jmb.2005.09.066

[19] Sass A. M. Schmerk C. Agnoli K. Norville P. J. Eberl L. Valvano M. A. Mahenthiralingam E. (2013) The unexpected discovery of a novel low-oxygen-activated locus for the anoxic persistence of *Burkholderia cenocepacia*. ISME J. 7, 1568–1581 23486248 10.1038/ismej.2013.36 PMC3721108

[20] Bradford M. M. (1976) A rapid and sensitive method for the quantitation of microgram quantities of protein utilizing the principle of protein-dye binding. Anal. Biochem. 72, 248–254 942051 10.1016/0003-2697(76)90527-3

[21] Crack J. C. Le Brun N. E. Thomson A. J. Green J. Jervis A. J. (2008) Reactions of nitric oxide and oxygen with the regulator of fumarate and nitrate reduction, a global transcriptional regulator, during anaerobic growth of *Escherichia coli*. Methods Enzymol. 437, 191–209 18433630 10.1016/S0076-6879(07)37011-0

[22] Hoang T. T. Karkhoff-Schweizer R. R. Kutchma A. J. Schweizer H. P. (1998) A broad host range Flp-FRT recombination system for site-specific excision of chromosomally located DNA sequences: application for isolation of unmarked *Pseudomonas aeruginosa* mutants. Gene 212, 77–86 9661666 10.1016/s0378-1119(98)00130-9

[23] Martínez-García E. de Lorenzo V. (2011) Engineering multiple genomic deletions in Gram-negative bacteria: analysis of the multi-resistant antibiotic profile of *Pseudomonas putida* KT2440. Environ. Microbiol. 13, 2702–2716 21883790 10.1111/j.1462-2920.2011.02538.x

[24] Miller J. H. (1972) Experiments in Molecular Genetics, pp. 352–355, Cold Spring Harbor Laboratory, Cold Spring Harbor, NY

[25] Crack J. C. Stapleton M. R. Green J. Thomson A. J. Le Brun N. E. (2013) Mechanism of [4Fe-4S](Cys)_4_ cluster nitrosylation is conserved among NO-responsive regulators. J. Biol. Chem. 288, 11492–11502 23471974 10.1074/jbc.M112.439901 PMC3630887

[26] Bates D. M. Popescu C. V. Khoroshilova N. Vogt K. Beinert H. Münck E. Kiley P. J. (2000) Substitution of leucine 28 with histidine in the *Escherichia coli* transcription factor FNR results in increased stability of the [4Fe-4S]^2+^ cluster to oxygen. J. Biol. Chem. 275, 6234–6240 10692418 10.1074/jbc.275.9.6234

[27] Crack J. C. Green J. Thomson A. J. Le Brun N. E. (2012) Iron-sulfur cluster sensor-regulators. Curr. Opin. Chem. Biol. 16, 35–44 22387135 10.1016/j.cbpa.2012.02.009

[28] Crack J. C. Green J. Hutchings M. I. Thomson A. J. Le Brun N. E. (2012) Bacterial iron-sulfur regulatory proteins as biological sensor-switches. Antioxid. Redox Signal. 17, 1215–1231 22239203 10.1089/ars.2012.4511 PMC3430481

[29] Smith L. J. Stapleton M. R. Fullstone G. J. Crack J. C. Thomson A. J. Le Brun N. E. Hunt D. M. Harvey E. Adinolfi S. Buxton R. S. Green J. (2010) *Mycobacterium tuberculosis* WhiB1 is an essential DNA-binding protein with a nitric oxide-sensitive iron-sulfur cluster. Biochem. J. 432, 417–427 20929442 10.1042/BJ20101440 PMC2992795

[30] Crack J. C. Smith L. J. Stapleton M. R. Peck J. Watmough N. J. Buttner M. J. Buxton R. S. Green J. Oganesyan V. S. Thomson A. J. Le Brun N. E. (2011) Mechanistic insight into the nitrosylation of the [4Fe-4S] cluster of WhiB-like proteins. J. Am. Chem. Soc. 133, 1112–1121 21182249 10.1021/ja109581t PMC3117330

[31] Hidalgo E. Bollinger J. M. Jr. Bradley T. M. Walsh C. T. Demple B. (1995) Binuclear [2Fe-2S] clusters in the *Escherichia coli* SoxR protein and role of the metal centers in transcription. J. Biol. Chem. 270, 20908–20914 7673113 10.1074/jbc.270.36.20908

[32] Ugidos A. Morales G. Rial E. Williams H. D. Rojo F. (2008) The coordinate regulation of multiple terminal oxidases by the *Pseudomonas putida* ANR global regulator. Environ. Microbiol. 10, 1690–1702 18341582 10.1111/j.1462-2920.2008.01586.x

[33] Zimmermann A. Reimmann C. Galimand M. Haas D. (1991) Anaerobic growth and cyanide synthesis of *Pseudomonas aeruginosa* depend on *anr*, a regulatory gene homologous with *fnr* of *Escherichia coli*. Mol. Microbiol. 5, 1483–1490 1787798 10.1111/j.1365-2958.1991.tb00794.x

[34] Kiley P. J. Reznikoff W. S. (1991) Fnr mutants that activate gene expression in the presence of oxygen. J. Bacteriol. 173, 16–22 1898918 10.1128/jb.173.1.16-22.1991 PMC207150

[35] Kovach M. E. Elzer P. H. Hill D. S. Robertson G. T. Farris M. A. Roop R. M. 2nd Peterson K. M. (1995) Four new derivatives of the broad-host-range cloning vector pBBR1MCS, carrying different antibiotic-resistance cassettes. Gene 166, 175–176 8529885 10.1016/0378-1119(95)00584-1

[36] Sharrocks A. D. Green J. Guest J. R. (1991) FNR activates and represses transcription *in vitro*. Proc. Biol. Sci. 245, 219–226 1684045 10.1098/rspb.1991.0113

[37] Gostick D. O. Green J. Irvine A. S. Gasson M. J. Guest J. R. (1998) A novel regulatory switch mediated by the FNR-like protein of *Lactobacillus casei*. Microbiology 144, 705–717 9534240 10.1099/00221287-144-3-705

[38] Wing H. J. Williams S. M. Busby S. J. (1995) Spacing requirements for transcription activation by *Escherichia coli* FNR protein. J. Bacteriol. 177, 6704–6710 7592457 10.1128/jb.177.23.6704-6710.1995 PMC177532

[39] Guan K. L. Dixon J. E. (1991) Eukaryotic proteins expressed in *Escherichia coli*: an improved thrombin cleavage and purification procedure of fusion proteins with glutathione *S*-transferase. Anal. Biochem. 192, 262–267 1852137 10.1016/0003-2697(91)90534-z

[40] Wong S. M. Mekalanos J. J. (2000) Genetic footprinting with mariner-based transposition in *Pseudomonas aeruginosa*. Proc. Natl. Acad. Sci. U.S.A. 97, 10191–10196 10963681 10.1073/pnas.97.18.10191 PMC27802

[41] Sievers F. Wilm A. Dineen D. Gibson T. J. Karplus K. Li W. Lopez R. McWilliam H. Remmert M. Söding J. Thompson J. D. Higgins D. G. (2011) Fast, scalable generation of high-quality protein multiple sequence alignments using Clustal Omega. Mol. Syst. Biol. 7, 539 21988835 10.1038/msb.2011.75 PMC3261699

[42] Kiefer F. Arnold K. Künzli M. Bordoli L. Schwede T. (2009) The SWISS-MODEL repository and associated resources. Nucleic Acids Res. 37, D387-D392 18931379 10.1093/nar/gkn750 PMC2686475

[43] Sayle R. A. Milner-White E. J. (1995) RasMol: Biomolecular graphics for all. Trends Biochem. Sci. 20, 374–376 7482707 10.1016/s0968-0004(00)89080-5

